# Identification of New Genetic Clusters in Glioblastoma Multiforme: *EGFR* Status and *ADD3* Losses Influence Prognosis

**DOI:** 10.3390/cells9112429

**Published:** 2020-11-06

**Authors:** Lara Navarro, Teresa San-Miguel, Javier Megías, Nuria Santonja, Silvia Calabuig, Lisandra Muñoz-Hidalgo, Pedro Roldán, Miguel Cerdá-Nicolás, Concha López-Ginés

**Affiliations:** 1 Department of Pathology, University of Valencia, 46010 Valencia, Spain; Lara.navarro@uv.es (L.N.); Javier.megias@uv.es (J.M.); Silvia.calabuig@uv.es (S.C.); lisandramh@gmail.com (L.M.-H.); Jose.m.cerda@uv.es (M.C.-N.); Concha.lopez@uv.es (C.L.-G.); 2 Department of Pathology, Hospital General Universitario Valencia, 46014 Valencia, Spain; nuriasantonja3@hotmail.com; 3 INCLIVA Foundation, 46010 Valencia, Spain; 4 Molecular Oncology Laboratory, Fundación para la Investigación Hospital General Valencia, 46014 Valencia, Spain; 5 Centro de Investigación Biomédica en Red de Cáncer, 28029 Madrid, Spain; 6 Department of Neurosurgery, Hospital Clínico Universitario Valencia, 46010 Valencia, Spain; Pedro.roldan@uv.es; 7 Department of Pathology, Hospital Clínico Universitario Valencia, 46010 Valencia, Spain

**Keywords:** glioblastoma, *IDH*, ADD3, EGFR, survival, high throughout techniques, precision

## Abstract

Glioblastoma multiforme (GB) is one of the most aggressive tumors. Despite continuous efforts to improve its clinical management, there is still no strategy to avoid a rapid and fatal outcome. *EGFR* amplification is the most characteristic alteration of these tumors. Although effective therapy against it has not yet been found in GB, it may be central to classifying patients. We investigated somatic-copy number alterations (SCNA) by multiplex ligation-dependent probe amplification in a series of 137 GB, together with the detection of *EGFR*vIII and FISH analysis for *EGFR* amplification. Publicly available data from 604 patients were used as a validation cohort. We found statistical associations between *EGFR* amplification and/or *EGFR*vIII, and SCNA in *CDKN2A, MSH6, MTAP* and *ADD3*. Interestingly, we found that both *EGFR*vIII and losses on *ADD3* were independent markers of bad prognosis (*p* = 0.028 and 0.014, respectively). Finally, we got an unsupervised hierarchical classification that differentiated three clusters of patients based on their genetic alterations. It offered a landscape of *EGFR* co-alterations that may improve the comprehension of the mechanisms underlying GB aggressiveness. Our findings can help in defining different genetic profiles, which is necessary to develop new and different approaches in the management of our patients.

## 1. Introduction

Glioblastoma multiforme, *IDH* wild-type (GB-*IDH*wt), is the most frequent malignant brain tumor in adults and the most aggressive in nature, with an average survival of around 15–18 months [1,2]. GB displays, in addition to morphological heterogeneity, a wide genetic heterogeneity [3,4]. This fact is, despite constant efforts, one of the main causes of the absence of effective treatment and thus, of the extremely poor prognosis this disease offers [5,6]. The last World Health Organization (WHO) classification tackles GB genetic heterogeneity, putting *IDH* status in the spotlight and associating it to prognosis [1,7]. The mutations that GB-*IDH*wt acquires during clonal evolution do not follow a linear development [4,8]. Many efforts have been made since The Pan Cancer project of TCGA deepened on the high frequency of alterations in the receptor tyrosine kinase/PI3K/PTEN/AKT/mTOR-signaling pathway (present in 88% of GB cases), along with the p53/MDM2/p14ARF molecular pathway (87%) and the CDKN2A/CDK4/6/retinoblastoma signaling pathway (78%) [1,9,10,11,12]. Those alterations seem to be a core requirement for GB pathogenesis and they are associated with a poor prognosis [13,14]. Identifying genetic changes in shared nodes of convergence may improve our understanding of diseases [4]. In addition, the identification of differential targets among GB *IDH*-wt genetic subgroups could lead to reach better approaches to GB management. *EGFR* amplification is a hallmark of glioma pathogenesis [4,15,16]. In the last decade, many molecules have shown promising results on pre-clinical or phase 1/2 trials targeting *EGFR*. However, they tend to fail, as recently happened with ABT-414 conjugated with ABBV-321 [16,17]. It is also disappointing that while TKI therapies have been successfully expanded to multiple types of cancer, none of them have improved the lifespan of GB patients [18]. Nevertheless, the interest of assessing *EGFR* copy number status [19,20,21,22,23,24] along with variants that have been related to cell proliferation, angiogenesis and invasion, and thus, with shortened survival, such as the *EGFR* variant III (*EGFR*vIII) [25,26,27], is undeniable.

Huge efforts have been made using high-throughput techniques for the genomic analysis of GB [4,28,29]. However, the complexity of the data they provide makes it difficult to draw a targetable GB portrait [1,28]. There is still a long way to progressively introduce objective biomarkers towards precision medicine adapted to the molecular profile of the tumor. High-throughput user-friendly techniques such as multiplex ligation-dependent probe amplification (MLPA) could be optimal for the development of new personalized therapies [24,30].

The main goal of this work was to characterize the frequent alteration of *EGFR* via its amplification or the presence of *EGFR*vIII in a series of 137 primary GBs and to assess concomitant somatic copy number alterations (SCNA). We focused on primary, de novo, *IDH*wt GBs, and we delved deeper into the potential use of MLPA to study formalin-fixed paraffin-embedded (FFPE) GB. We aimed to define networks of genetic alterations from a simplified point of view, compared to high-throughput techniques, using such a common kind of material as paraffin embedded tumor tissue samples are. Our results highlight the existence of three groups of tumors according to different complex genetic profiles, where *EGFR* plays a crucial role. We also present the importance of *ADD3* and *EGFR*vIII as genetic biomarkers that may determine a more accurate prognostic for GB patients. Taken together, our results improve the comprehension of the mechanisms underlying GB aggressiveness and establish a new genetic classification that could enhance the clinical management of GB patients.

## 2. Materials and Methods

### 2.1. Patients, Samples and Clinical Study

GB samples were obtained from 137 patients surgically treated at the Clinic Hospital of Valencia between 1995 and 2010, with known follow-up. The study was reviewed and approved by Institutional Ethics Committee of the University of Valencia and Clinic Hospital of Valencia. Tumor specimens were fixed in neutral buffered formalin, embedded in paraffin, sectioned, and stained with hematoxylin and eosin (H&E). Samples were categorized according to the WHO classification [1]. Glioblastoma, *IDH*-mutant (n = 9), including those that progressed from lower-grade gliomas, were not included in the different statistical analyses. All patients underwent treatment consisting of maximum safe tumor resection and none of them received chemotherapy or radiotherapy before surgery. Similar schemes of first-line treatment, encompassing radiotherapy (50–65 Gray) with concomitant and adjuvant temozolomide-based chemotherapy were applied after surgery in all the cases. A retrospective survival analysis was performed. Overall survival (OS) was calculated as time from surgery to death. Event times were censored if the patient was alive at the time of last follow-up.

### 2.2. DNA Extraction, Molecular Analysis of IDH1/2, TP53 and MLPA

Genomic DNA was extracted from FFPE tissue samples using a QIAamp DNA FFPE Tissue Kit (Qiagen, Inc., Valencia, CA, USA) according to the manufacturer’s instructions. We analyzed by direct sequencing the genomic regions spanning wild-type R132 of *IDH*1 and wild-type R172 of *IDH*2. Exons 5–8 of TP53 were also sequenced. PCRs were performed using standard buffer conditions, 200 ng of DNA and an AmpliTaq Gold Master Mix (Thermo Fisher Scientific, Waltham, MA, USA). PCR products were purified with Centricon columns (Amicon, Beverly, MA, USA) and analyzed on an ABI 310 Sequencer (Applied Biosystems, Foster City, CA, USA). Primer sequences forward (fw) and reverse (rv) were as follow: *IDH*1 fw 5′-ACCAAATGGCACCATACGAA and rv 5′-TCACATTATTGCCAACATGACTT, *IDH*2 fw 5′-CCAATGGAACTATCCGGAAC and rv 5′-CCTCTCCACCCTGGCCTAC, TP53 (exon 5) fw 5′-CAGCCCTGTCGTCTCTCCAG and rv 5′-TTCAACTCTGTCTCCTTCCT, TP53 (exon 6) fw 5′-GTCTGGCCCCTCCTC AGCAT and rv 5′-GTCTGGCCCCTCCTCAGCAT, TP53 (exon 7) fw 5′-CTCATCTTGGGCCTGTGTTA and rv, 5′-AGTGTGCAGGGTGGCAAGTG, TP53 (exon 8) fw 5′-ACCTGATTTCC TTACTGCCTCTTGC and rv 5′-GTCCTGCTTGCTTACCTC GCTTAGT.

Multiplex Ligation-dependent Probe Amplification (MLPA) using the MLPA KIT P105 (version C1-C2) and ME024 (version A1-B1) was performed in accordance with the manufacturer’s protocol (MRC Holland, Amsterdam, Netherland) [30,31]. Amplification products were separated on an ABI 310 Sequencer (Applied Biosystems, Inc, Foster City, CA, USA) and data analysis was made with Coffalyser excel-based software (MRC-Holland) [32], where relative probe values of probe-amplified products are compared with normal controls. These kits included multiple glioma and cancer related genes that were evaluated depending on their probes based on previous reports [30,31,33,34], as explained below. Both kits are approved for investigational-use only.

### 2.3. Status of EGFR: EGFRvIII, Copy Number Alterations and Interphase Fluorescence In Situ Hybridization

*EGFR* was studied by MLPA using the P105 kit (MRC-Holland). It includes 11 probes for exons 1–8, 13, 18 and 24 of EGFR. This design allowed us to determine on one hand, the presence of the variant III and on the other hand, somatic copy number alterations (SCNAs) of *EGFR* in the samples analyzed. Following previously published descriptions, to identify *EGFR*vIII we determined the average value for exons 2–7 probes and established the ratio with the average value of probes for exons 1, 8, 13, 18 and 24. Patients with ratios below 0.8 were considered to harbor the *EGFR*vIII [31,35]. SCNAs of *EGFR* were determined based on the average value of exons 1, 8, 13, 18 and 24 in order to exclude the ones that are frequently involved in EGFR variants. The thresholds applied classified the samples as no amplified (0.7 <  x  <1.3) or gained (x ≥ 1.3) for downstream statistical analysis. We also analyzed the EGFR gene status in interphase cells by dual-color FISH probes using interphase Fluorsence in situ hybridization (iFISH). For that purpose, we used tissue microarrays (TMAs) and the probe LSI EGFR SpectrumOrange/CEP-7 SpectrumGreen Probe from Vysis (Abbott Laboratories, IL, USA). Hybridizations were performed according to the manufacturer’s instructions, and signals were counted in two different regions of 200 non-overlapping nuclei. We calculated the ratio between the average signal count of *EGFR* and the control probe CEP-7 (*EGFR*/CEP7 ratio). *EGFR* was considered to be amplified when the EGFR/CEP-7 signal ratio was >2 [36]. Cases were subclassified according to previous descriptions as GBs with high level of amplification (H-amp) when more than 20% of the cells showed more than 20 copies of EGFR. GBs with low levels of amplification (L-amp) included cases with 5–20% of cells with 4–12 copies of EGFR. Cases without amplification (N-amp) showed two copies of EGFR. The exact ratio was not calculated in cases with high amplification levels [19,36]. The aim of this validation study was to determine the concordance rates of these techniques. Concordance between both techniques was assessed by the Cohen’s Kappa statistic. It has a range of 0–1.0 and κ values <0.2 means a poor agreement, 0.21 < κ < 0.4 means a weak agreement, 0.41 < κ < 0.6 means a moderate agreement, 0.61 < κ < 0.8 means a good agreement and 0.81 < κ < 1 means a very good agreement between techniques.

### 2.4. Analysis of Locus 9p21 and Other Glioma and Cancer-Related Genes

SALSA MLPA P105 and ME024 included a collection of probes to determine *PTEN* status and genes located on 9p21. Many probes in order to characterize *CDKN2A-CDKN2B* (p15INK4B-p14ARF-p16INK4A) were included. These kits also comprised probes for other genes located up and downstream on 9p21.3 (*KLHL9, MIR31, MLLT3* and *MTAP*) and for genes located near on 9p (*DOCK8* 9p24.3 and *GLDC* on 9p24.1, and *PAX5* on 9p13.2). In addition, a wide collection of probes addressed to many different genes were assayed, including: *DYSF* (2p13), *MSH6* (2p16), *SIX3* (2p21), *SPAST* (2q22.3), *EDAR* (2q12.3), *CTNNB1* (3p22), *ATR* (3q23), *TGFBR2* (3p24), *CFI* (4q25), *WDR36* (5q22.1), *IL4* (5q31), *CDH7* (8q12), *ADD3* (10q25.2), *MEN1* (11q13.1), *SMPD1* (11p15.4), *COL2A1* (12q13.11), *HNF1A* (12q24), *PCCA* (13q32.3), *ING1* (13q34), *ATL1* (14q22.1), *SNRPN-HB2-85* (15q11.2), *SPG11* (15q21.1), *IQGAP1* (15q26.1), *MVP* (16p11.2), *TRAF4* (17q11.2), *ERBB2* (17q12) *NPC1* (18q11.2), *CACNA1A* (19p13.2), *SMARCA4* (19p13.2), *JAG1* (20p12.2), *ADAMTS5* (21q21.3), and *TIMP3* (22q12.3). The variety of loci explored offered a detailed landscape of GB SCNAs.

To categorize MLPA call to SCNA value, we established deletions as x < 0.7, normal as 0.7 < x < 1.3 and gain as x > 1.3. When probe values within the different exons of one locus were heterogeneously distributed across some categories such as heterozygous and homozygous deletions, we defined the category in which more than 70% of probe values belonged. For convenience, homozygous and/or heterozygous deletions collectively were referred to as deletion, while amplification and/or gain as gain/amp. Non-canonical SCNAs such as gain/amp in *CDKN2A*, *PTEN* or *TP53* and deletion in *EGFR or CDK4* were not considered in the data analysis.

### 2.5. Statistical Analysis

The statistical analysis of the data was carried out according to the type of variable. Quantitative variables were evaluated using the Kolmogorov-Smirnov and Levene tests; depending on their results and their characteristics, Student’s t-test, ANOVA, Mann–Whitney–Wilcoxon test or Kruskal–Wallis test were performed. For comparisons among categorical variables, Fisher’s exact, Pearson’s chi-squared, and Kruskal–Wallis test were used depending on the number or rows/columns and the expected frequencies. A survival analysis using the Kaplan–Meier method was also done. The statistical significance of these curves was calculated using the log-rank (Mantel–Cox) test. Significance was accepted at least at *p* < 0.050 level. Data were analyzed with SPSS (version 26) software (IBM, Madrid, Spain). To perform the clusters, the average-linkage method was used. Therefore, distance between clusters was obtained as the average distance between all possible pairs of cases from both clusters, providing robust groups. Euclidean distance was used because of the binary scale of the genetic variables. The choice of three clusters was decided from the hierarchical tree, according to the level of heterogeneity at which the clusters were combined. Cluster combination stopped when this level was roughly 70% of the total amount.

### 2.6. TCGA Analysis and Functional Protein Associations

We accessed data for GB samples from The Cancer Genome Atlas (TCGA) by using cBioPortal for Cancer Genomics (www.cbioportal.org) [12,37,38] to validate whether the SCNAs detected in our cohort were associated with EGFR amplification there. We studied the Genomic Profile “Putative copy-number alterations from GISTIC” for the latest dataset available in cBioportal for GB (TCGA, Provisional 604 samples). Copy number alteration data from 577 cases were obtained and further analyzed. A “User-Defined List”, including *EGFR, CDKN2A, MTAP, TRAF4, JAG1* and *MSH6*, was entered into the “Enter Gene” box. Samples were classified for each gene according to their putative copy number variation calculated by GISTIC with default cBioportal thresholds 33. The groups were Diploid (0), Shallow Deletion (−1), Deep deletion (−2), Gain (1) and Amplification (2). The associations between EGFR amplification and the copy number profile were analyzed using the “Plots” tool and retrieving the raw data. Outlier values (n < 10 from 577 cases) were negligible. Statistical significance for amplification vs. diploid and deletion (shallow or deep) vs. diploid for each gene was assessed using Fisher’s exact test.

We also used the Search Tool for the Retrieval of Interacting Genes/Proteins (STRING) database of known and predicted protein-protein interactions (https://string-db.org) to establish associations among the proteins encoded by the genes affected by SCNAs in this series. This database contains information from numerous sources, including KEGG, Reactome or Pubmed, among others [37]. To make a restrictive analysis, we explored both evidence and confidence meaning of network edges and we increased the minimum required interaction score to ‘high confidence (0.700)’ to reach a greater reliability.

## 3. Results

### 3.1. Clinical and Histopathological Data

**Figure 1:**
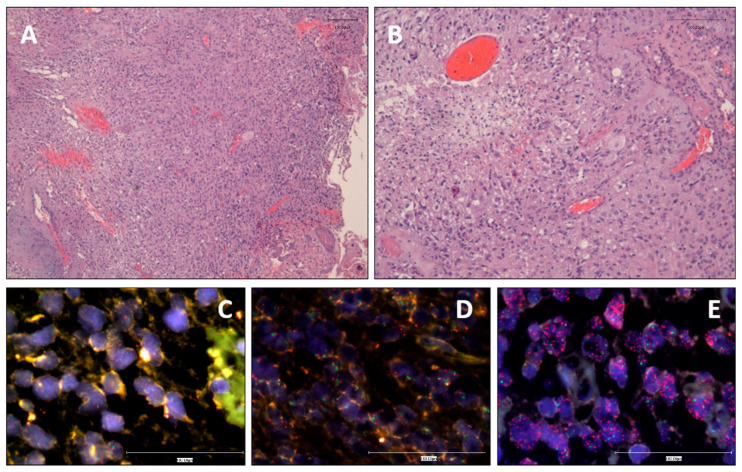
Representative microphotographs of GB, *IDH-*wt. (**A**) High cellularity and poor differentiation with prominent microvascular proliferation and necrosis (Hematoxilin and Eosin, 10×). (**B**) Highly anaplastic, heterogeneous cells and microvascular proliferation (Hematoxilin and Eosin, 20×). (**C**–**E**) iFISH microphotographs showing the status of *EGFR* by an orange LSI-EGFR probe and the centromere of chromosome 7 as a reference, stained by CEP-7 green probe in cases representing the tree levels of amplification. (**C**) No *EGFR* amplification (40×). (**D**) Low level of *EGFR* amplification (40×). (**E**) High *EGFR* amplification (40×).

Our study included 137 patients. Clinical data, including age, sex, tumor location, size, initial symptom and Karnofsky performance status scale (KPS), are summarized in Table 1. Mean age at diagnosis was 57.7 years and male/female ratio was 1.17. OS was 210 days and did not reflected statistical differences depending on sex nor age. Histologically, all tumors showed features of GB with pleomorphic astrocytic tumor cells, prominent microvascular proliferation and necrosis (Figure 1). From confirmed primary GB (n = 135), the majority were IDHwt (n = 128) but a little subgroup of tumors displayed IDH mutations (n = 7) despite being primary GB (GB-IDHmut). In agreement with the WHO 2016 classification, these GB-IDHmut-affected patients were significantly younger (40.9 years in IDHmut vs. 59 years in IDHwt, *p* < 0.001 **). OS was also significantly higher in IDHmut than in IDHwt patients (3300 days vs. 180 days, *p* < 0.001 **). From the 128 cases, 63.9% of patients were >55 years old at diagnosis, the 56.3% were male and 76.9% had a KPS ≤ 85 preoperatively. Interestingly, tumor Pearson correlation test demonstrated an association between tumor size and OS (*p* = 0.020 *).

### 3.2. EGFR Status Assessment by iFISH and MLPA

iFISH analysis showed a distribution of cases among N-amp, L-amp and H-amp of 35.6%, 12.7% and 51.7%, respectively (Figure 1). Similarly, MLPA analysis, which only separates two categories, showed that 29.7% of cases had no EGFR gains, whereas 70.3% of the cases displayed gain of EGFR copies. Comparing FISH H-amp and L-amp with MLPA “gain”, and FISH N-amp with MLPA “no gain”, concordance between both techniques was good, with a coincident result in 83.05% of the samples (Cohen’s kappa index: 0.610). Agreement was 93.4% for L-amp and H-amp GBs assessed by iFISH that were detected as gains by MLPA. Regarding N-amp tumors, the concordance dropped to 64.3% (Supplementary Table S1 shows the result from both techniques for each case). The visual determination of EGFR made iFISH more suitable to categorizing the cases according to their amplification status. Thus, the following analyses regarding EGFR amplification status were established according to the iFISH data. Among the 128 GB patients, we found no significant associations between EGFR amplification and survival (*p* = 0.387). Clinical data did not reveal any strong association to the amplification status either: 50.8% of men and 49.2% of women displayed H-amp EGFR, reflecting no differences depending on the basis of sex. We did not find significant associations between EGFR amplification and patient age.

### 3.3. EGFR Variant III Is More Frequent in Women and Is Associated with Shortened Survival

**Figure 2:**
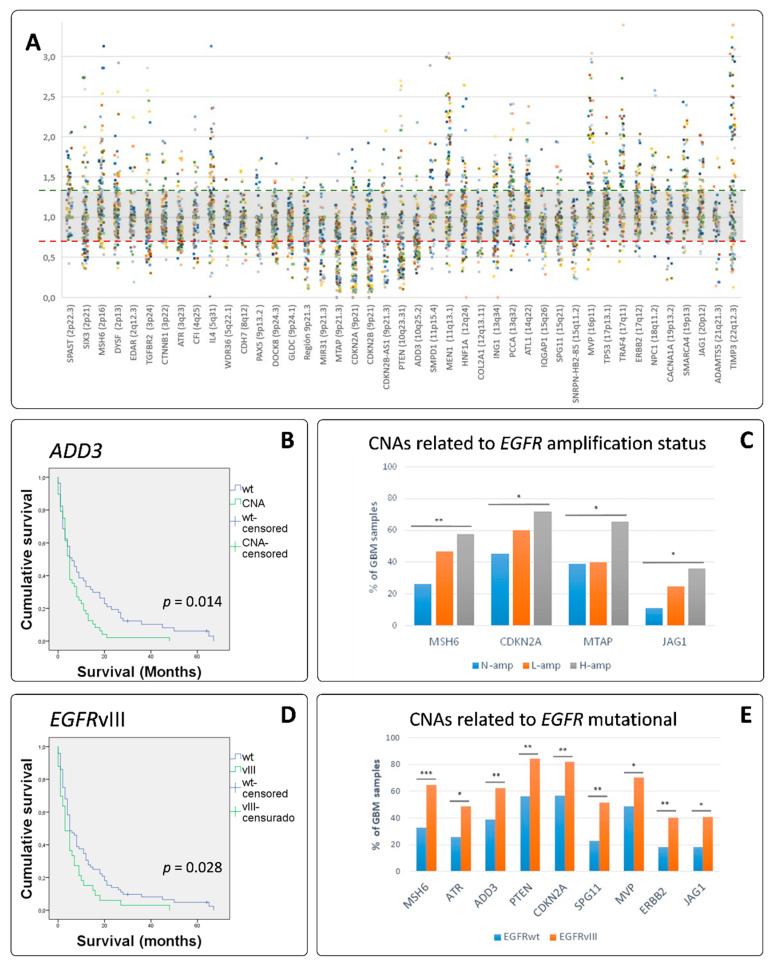
GB somatic copy number alterations (SCNA). (**A**) Distribution of MLPA calls from each case on the targeted genes assayed showing the heterogeneity of GB. (**B**) Association between ADD3 SCNA and overall survival (OS). The Y-axis represents the cumulative survival time in terms of probability: it oscillates between 0 (0% of cases) and 1 (100% of cases). The X-axis shows the survival period, expressed in months. The blue line represents survival on patients with wild-type ADD3 (mean = 13.46 months). The green line represents survival in patients which tumors showed SCNA on ADD3 (mean = 6.98 months). Long Rank (Mantel–Cox) analysis demonstrate statistical significance. (**C**) Concomitant SCNA with the EGFR amplification status. MSH6 *p* = 0.007, CDKN2A *p* = 0.023, MTAP *p* = 0.017 and JAG1 *p* = 0.029. (**D**) Association between EGFRvIII and OS. The blue line represents survival on patients with EGFRwt (mean= 180 days). The green line represents survival in patients which tumors showed EGFRvIII (mean= 150 days). Long Rank (Mantel–Cox) analysis demonstrate statistical significance. (**E**) Concomitant SCNA with the presence of EGFRvIII. MSH6 *p* = 0.001, ATR *p* = 0.019, ADD3 *p* = 0.010, PTEN *p* = 0.002, CDKN2A *p* = 0.004, SPG11 *p* = 0.003, MVP *p* = 0.032, ERBB2 *p* = 0.008, and JAG1 *p* = 0.013. * was used for 0.050 > *p* > 0.010, ** for 0.010 > *p* > 0.001 and *** for *p* ≤ 0.001.

MLPA allowed us to determine the presence of EGFRvIII. IDHwt GBs (n = 128) exhibited EGFRvIII in 35.2% of the cases (n = 45). Clinical data revealed no differences between both groups regarding age, tumor location nor size. Nevertheless, there was a significant increase in the presence of this variant in women (55.6% EGFRvIII vs. 37.3% EGFRwt, *p* = 0.047 *). Interestingly, Kaplan–Meier analysis revealed statistical differences in OS (Figure 2, *p* = 0.014 *), accounting 150 days in EGFRwt vs. 90 days in EGFRvIII (Student t-test *p* = 0.027 *). In addition, EGFRvIII was significantly more frequent in GBs with EGFR amplification. We found it in 10 cases of N-amp GBs (23.8%), 2 cases of L-amp GBs (13.3%) and 31 cases of H-amp GBs (50.8%, *p* = 0.003 **).

### 3.4. MLPA Analysis Showed a Great Heterogeneity in GB

We identified that 100% of the tumor cases showed SCNA in at least two of the genes analyzed. In addition, from all the loci explored, we found SCNA in all of them in at least seven cases. An overview of the prevalence of genetic alterations identified in 128 GB-IDHwt patients is shown in Table 1. A summary of all the SCNAs detected among the studied genes can be seen in Figure 2A which offers a landscape of the high genetic heterogeneity found. We detected alterations affecting more than 45% of the cases on EGFR (70.1% of the cases), CDKN2A (65.6%), TIMP3 (64.1%), MEN1 (57.8%), CDKN2B (56.3%), MVP (56.3%), PTEN (54.8%), MTAP (53.5%), ADD3 (46.9%) and PCCA (46.6%). From those loci, only ADD3 tend to be more altered in women (55.4% of the cases) than in men (40.3% of the cases). However, ADD3 demonstrated to be associated with OS, as patients with SCNAs on ADD3 showed an OS of 6.98 ± 1.17 months while patients with no-SCNA on ADD3 showed an OS of 13.46 ± 2.24 months (*p* = 0.012 *). Long Rank (Mantel–Cox) analysis demonstrate a statistic association (*p* = 0.014 *, Figure 2B).

### 3.5. EGFR Amplified GBs Displayed Different SCNAs to Non-EGFR Amplified Cases

In this series, four genes revealed statistical differences on their affectation, depending on the amplification status of EGFR: losses/gains of MSH6 on 2p16.3, losses of CDKN2A and MTAP, both on 9p21 and gains of JAG1 on 20p12.2 (Figure 2C). TCGA analysis through cBioportal supported our data, as it showed strong associations between both CDKN2A and MTAP losses and EGFR gain/amp (*p* < 0.0001 ***). Of note is that both genes are located in 9p21. TCGA data also showed statistical association between JAG1 gain/amp and EGFR gain/amp (*p* < 0.0001 ***). MSH6 showed alterations in a little number of GBs and did not reach a significant result (Table 2). STRING analysis provided a PPI enrichment *p*-value of 0.000987 and association to NOTCH3 activation and to a negative regulation of cell–matrix adhesion. Based on previous reports [7], we looked for GB with triple SCNA (EGFR, CDKN2A and PTEN); in our series, this appeared in 25.0% of the cases and they showed an OS of 7.64 ± 1.97 months, which was lower than the 11.25 ± 1.71 months in cases with no triple SCNA.

Looking for differences depending on EGFR status, we found in our series that EGFRvIII cases displayed statistically different SCNAs than their EGFRwt counterparts: MSH6 (2p16.3), ATR (3q23), ADD3 (10q25.1), PTEN (10q23.31), CDKN2A (9p21), SPG11 (15q21), MVP (16p11.2), ERBB2 (17q12), JAG1 (20p12.2) (Figure 2E). STRING functional analysis of these genes revealed a PPI enrichment *p*-value of 9.2 × 10^−6^ and a non-random association with negative regulation of cell–matrix adhesion processes (false discovery rate –FDR- of 0.00100).

### 3.6. Clustering Analysis Revealed Different Genetic Glioblastoma Groups

**Figure 3:**
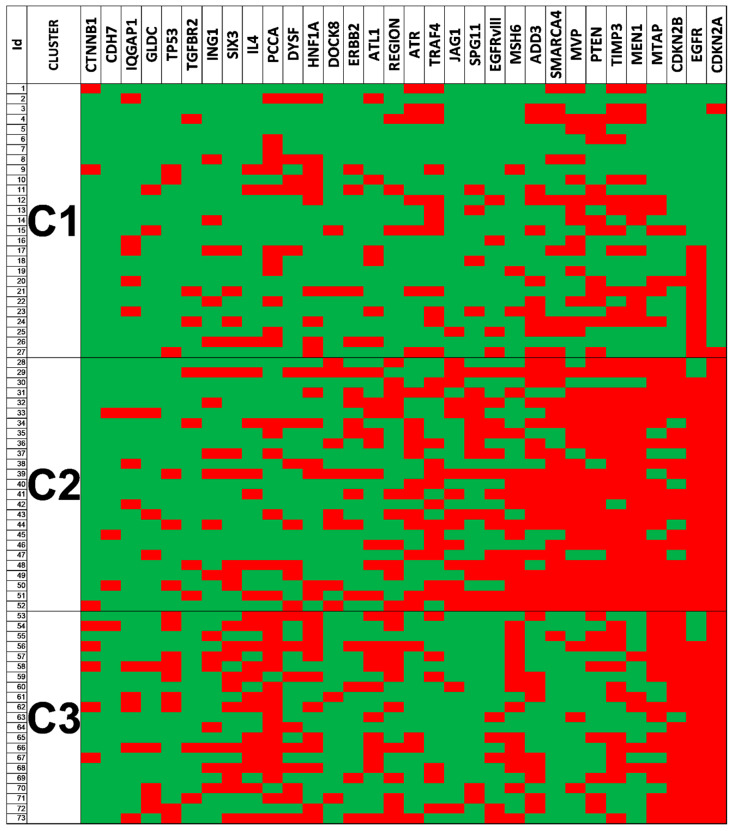
Clustering analysis. Heatmap from the hierarchical cluster classification. The patients included are represented on the Y-axis and the genes that contributed to the model on the X. Green squares show no SCNA, red squares show SCNA. C1, C2 and C3 are shown separately, and the distribution of SCNAs among offers a landscape where C1 is the one with less genetic alterations while C2 is the most affected one.

Hierarchical cluster classification distinguished three groups (C1, C2 and C3) depending on the frequencies of alteration within the different loci explored (Table 3). In this analysis we excluded 37 patients because some markers were not available. From the 91 patients to classify, 18 were unclassifiable subjects due to the diversity of the SCNAs found and 73 were distributed among the three different groups performed. The clusters showed partially overlapped changes and others completely differentiated among them (Figure 3). The Chi-squared test was used to assess the dependence between the normal/altered presence of a gene and the membership cluster in order to identify which genes have a higher power of discrimination. When the expected frequency in cells of the cross-table was too small (n < 5) in more than 33% of cells, the Kruskal–Wallis test was used as an alternative to Chi-squared. Regarding clinical data, size was similar in the different clusters, showing an average of 5.7 cm in C1, and 5.3 cm in C2 and 4.9 in C3. Age at diagnosis also showed similar averages (59, 57 and 61 years, respectively). Overall survival was 7.2 months for C1, 5.9 for C2 and 10.7 months for C3. Genetically, the analysis showed that C1 was the least affected, C2 showed alterations of near half of the loci explored in more than 50% of the cases, and C3, displayed an intermediate situation, with near 30% of the genes included affected in more than 50% of the cases. When we analyzed the EGFR amplification status by iFISH in relation to these clusters, we found statistically significant differences (*p* = 0.007 **): most cases from C1 were N-amp (63.0%), compared to 28.0% and 19.0% in C2 and C3, respectively. Most cases from C2 and C3 where H-amp (64.0% and 61.9%, respectively), compared to 22.2% in C1. This distribution of the cases for the L-amp group was more homogeneous, accounting 14.8%, 8.0% and 19.0% in C1, C2 and C3, respectively.

### 3.7. Genetic Changes According to Clustering Analysis Point to Differentially Altered Pathways

**Figure 4:**
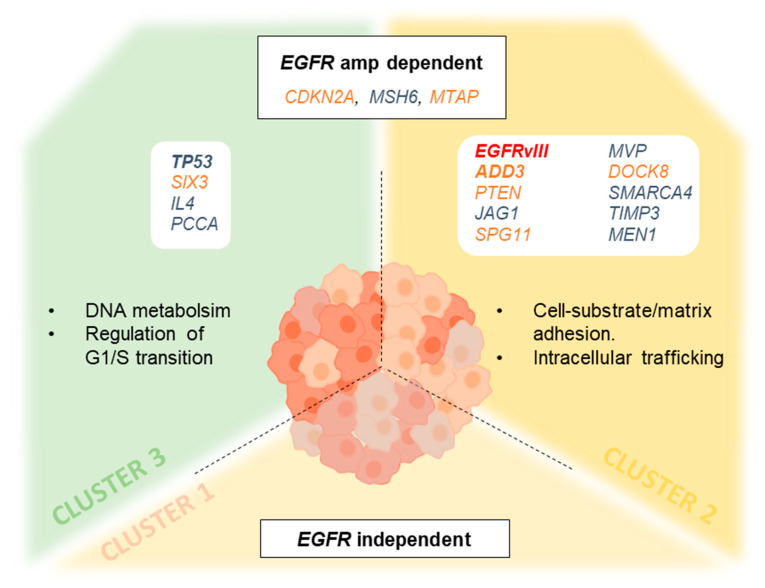
Pathways outlined by clustering analysis. The introduction of genes that differentially contributed to the clusters on the STRING database analysis platform offered a variety of genetic pathways and processes that were especially damaged in C2 and C3. Genes in blue displayed gains, genes in orange displayed losses, *EGFR*vIII includes loss from exons 2–7. Genes in bold were the most distinctive among the clusters. It needs to be mentioned that MSH6, in addition to gains, showed losses in <10% of the cases. The main difference between C2/C3 and C1 is that on C1, *EGFR* alterations were found only in half of the cases. Despite C2 and C3 share some alterations, the downstream connections lead to a subtly quickest disease on C2 compared to the slowest situation on C3.

EGFRvIII and losses in ADD3, associated with survival, were concentrated in cluster 2. In concordance, C2 displayed as aforementioned, the shortest OS. In addition, it showed the highest frequency of SCNAs in CDKN2A, MEN1, EGFR, TIMP3, PTEN, MTAP, MVP, SMARCA4, ADD3, MSH6, JAG1, SPG11 and DOCK8. The gene function and pathway annotation analysis by the STRING software showed a PPI enrichment p-value of 0.000522 and an association to the biological processes’ ‘regulation of cell-substrate adhesion’ (FDR = 8.36 × 10^−5^, by JAG1, CDKN2A, PTEN and MEN1) and ‘cell–matrix adhesion’ (FDR = 0.000522, by JAG1, CDKN2A and PTEN). Moreover, the Cellular Component analysis showed a significant association to the presence of the proteins encoded in different parts of the cell. They were detected both in the plasma membrane region (count in gene set: 5/1061), in organelle lumen (count in gene set: 10/5162) and in the nuclear part (count in gene set: 9/4359), all with FDR = 0.0364, suggesting an intense trafficking of these proteins through the cell (Figure 4).

Cluster 3, in common with C2, showed losses in CDKN2A in 100% of the cases, a high frequency of EGFR amplification and SCNA in MTAP and MSH6. Additionally, this C3 displayed frequent SCNAs in TP53, IL4, PCCA and SIX3. STRING analysis showed a PPI enrichment *p*-value of 0.0019 and an association to the biological processes ‘regulation of cell cycle phase transition’ with an FDR of 0.0060. However, no specific cellular component was revealed. Finally, C1 was the less altered cluster. It showed a statistically significant lower level of CDKN2A, MSH6, MTAP and EGFR alterations compared with its counterparts.

Overall, GB-IDHwt displayed a wide genetic heterogeneity. However, cluster analysis allowed a separation based on the frequency of alterations detected by MLPA into three groups. From them, the two that displayed EGFR amplification offered completely different outcomes, depending on the presence of additional alterations, as SCNA on ADD3 and the variant III of EGFR. These two changes were shown to be independent biomarkers for bad prognosis, and their statistical association to C2 highlights the interest of exploring the aggregation of genetic alterations in it.

## 4. Discussion

*EGFR* genetic alteration is an essential component of the portrait of most GBs occurring in 57% of tumors [4,38]. This frequency is similar to what we find for *EGFR* amplification and *EGFR* mutation in this work. This fact, along with the devastating outcome of GB, justifies the continuous search for associations in relation to *EGFR* changes and the therapeutic responses of patients. In light of the TCGA project, RTK alterations and their downstream effectors are of potential interest as targetable driver mutations [39]. However, it could be especially interesting to be able to classify our patients depending on specific common changes or even better, different pathways affected, to understand what makes them undergo a quicker or slower disease.

The use of iFISH to precisely determine the *EGFR* amplification status is the current gold-standard in GB [19,40]. Previous works of our and others groups, demonstrated that this method allows the detection of intermediate levels of amplification [19,24]. Lassman and his colleagues deepened the potential of different new techniques in comparison to iFISH with positive results [40]. In the present work, we compared the potential of MLPA to that aim because it is a user-friendly and cost-effective technique, and the Cohen agreement we got was also good. The point of iFISH remains the possibility of separating that intermediately amplified group, for which it is not entirely clear whether it represents a progression via the amplification status or a separate path for tumor progression in GB. In any case, MLPA proves to be an easy and fast technique, with the additional advantage of being able to determine mutant variants such it is *EGFR*vIII, in agreement with previous works [21,24,31,35].

The wide heterogeneity of cancer cells is a common challenge in terms of learning how to stop tumor growth. The clonal evolution of GB *IDH*wt and the acquisition of new mutations represent a major problem for finding effective therapies [1,4,8,23]. This heterogeneity is patent in our work; from all the loci explored, we found SCNA in all of them in at least one case. The genetics of H-amp GB and GB displaying *EGFR*vIII offers landscapes that are statistically different from their N-amp/L-amp or *EGFR*wt counterparts, respectively. STRING analysis correlates the SCNA statistically associated with amplified-*EGFR* GBs with *NOTCH3* activation and negative regulation of cell–matrix adhesion. Crespo et al., using high-density (500K) single-nucleotide polymorphism arrays, achieved similar relevance for the axis established with *MTAP* [16]. In concordance with that work, our group of patients that displayed *EGFR*vIII, which is the one with the worst outcome, also showed the impairment of cell–matrix adhesion processes. Despite the alteration of different sets of genes, we found a higher frequency of *EGFR*vIII among the *EGFR* amplified GBs, in concordance with previous works [9,24], and interestingly, we found a significant association between *EGFR*vIII and a shortening in survival, supporting previous descriptions [22,27,41].

An outstanding finding when analyzing the genes whose alterations were associated with *EGFR*vIII is that *ADD3* SCNAs is associated with bad prognosis, with a marked reduction in OS. *ADD3* codifies the γ-adducin, which build heterotetramers with its counterparts α- and β-adducin, and has been widely studied in red-cell membranes [42]. Different works put a spotlight on adducin’s controversial role as either oncogene or tumor suppressor in cancer [42,43,44,45]. Our findings support a recent report of Kiang KM et al. that points to the downregulation of *ADD3* in GB, but not in less malignant gliomas, as a critical event during malignant progression [46]. While most cell-based studies suggest an oncogenic behavior, different papers on glioma tumor specimens, in agreement with our data, relate *ADD3* downregulation to progression [43,44] and to migration [45]. Other cancer that shows *EGFR* amplification, such as non-small cell lung cancer, shares this *ADD3* infraexpression associated with cell migration [47]. We suggest that the use of such easy techniques as MLPA to assess *ADD3* SCNA could be considered for diagnostic routines to better tailor clinical decisions as an independent prognostic factor and to delve deeper into the GB classification of patients.

The frequency of *ADD3* SCNAs and its co-occurrence with *EGFR*vIII led us to look for genetic clustering. Thus, according to the genetic probes analyzed by MLPA, clustering analysis causes GBs to separate into three different groups. The first cluster (C1) offers a low rate of SCNAs compared with the rest. Cluster 2 (C2) and cluster 3 (C3) share a high rate of SCNAs in *MTAP, CDKN2A, MSH6* and *EGFR*, in agreement with previous descriptions [16], and suggesting that GBs from both clusters display alterations in DNA metabolic processes (by STRING). However, the second cluster (C2) concentrates the co-existence of EGFR alterations through *EGFR*vIII or *EGFR* amplification and the highest rate of SCNAs. Of these, it is worth mentioning that JAG1, which is involved in angiogenesis [48], *MVP*, was recently implicated in vesicle trafficking [21] or the aforementioned *ADD3*. Coherent with these data, this cluster displayed the shortest OS. It is curious that MSH6, in addition to showing gains as was previously reported in association with resistance to chemotherapy [49], displays losses in a short set of cases in this series. These cases with losses do not show an increase on the tumor genetic burden consequence of the defect on DNA repair, but interestingly, they show a really subtle increase in survival. It is not significant, but it deserves to be further studied, as it resembles the protective effect of the promoter methylation of *MGMT*, which improves responsiveness to temozolomide treatment [1,50]. Regarding *MTAP*, its deficiency is usually seen as a collateral effect of *CDKN2A* deletion, because of its location next to this gene [51,52]. Nevertheless, it is necessary to improve our understanding of the consequences of this loss because it could offer different insights into therapy. It is known that the loss of *MTAP* influences the metabolism of ATP: both adenine and adenosine are disturbed as a consequence of the disruption of the polyamine salvage pathway [53,54]. This fact could be related to the metabolic reprogramming strategy to actively modulate the immune landscape of GB [55]. Contrary to what was expected, it was shown that adenosine did not significantly accumulate in GB [55]. This fact would be in line with previous descriptions of N6-isopentenyladenosine and other modified nucleosides, with important anti-proliferative and pro-apoptotic effects in GB cases that display amplification on *EGFR* [56,57]. Interestingly, in our study, cluster 2 and 3 showed amplifications on *EGFR* and losses on *CDKN2A* in all cases, along with a high proportion of cases displaying *MTAP* losses. It would be desirable to further study whether the efficacy of those molecules could represent a therapeutic benefit for these specific patients with adenosine metabolism disturbances.

A noteworthy fact is that *ADD3* and *EGFR*vIII, both previously reported as alterations associated with shortened survival in GB [27,46] and confirmed here, are a signature in this C2. On the other hand, C3 involves *TP53* alterations in addition to the shared ones. This fact completely makes sense considering that *TP53* mutations are, according to the WHO, more frequent in the group of GB with *IDH* mutations, characterized by a higher survival than *IDH*wt GBs [1]. Moreover, *EGFR*vIII has been broadly associated with a poorer outcome [22,27]. These findings reflect that, not only the amplification status of *EGFR* could be decisive in the comprehension of GB progression, but also the interconnection established with parallel genetic pathways.

A comparison between the main findings in the clusters defined here and the previously reported TCGA expression subtypes [10,38] is worthy of some comments: the small set of GB, *IDH*-mutant cases we studied may fit in the proneural TCGA group, with mutations in *TP53* in half of the samples and loss of *PTEN* in 2/3 of the cases. Similarly, lifespan is higher, as we expected for being *IDH1*-mutant [1]. Cluster 2 fits quite well in the “Classical” subtype [10,38]. *EGFR* amplification is the main characteristic accompanied by losses on *CDKN2A* in all the cases and losses on *PTEN* in a high proportion (88%). However, cluster 3 seems to be a variant of this classical subtype. It is also characterized by *EGFR* amplification and losses on *CDKN2A*, but losses on *PTEN* drop down to a half. However, other alterations define it better than *PTEN* status, such as gains on TP53 or losses on SIX3. It is worth mentioning that *TP53* stands out, but it does so because of SCNA, and not mutations, as would be more characteristic of the mesenchymal TCGA subtype [10,38]. Thus, between these two clusters, a better genetic definition of the patients is offered. It is of note that C1 seems to have little to do with *EGFR,* in contrast to C2 and C3. C1 agrees with the mesenchymal subtype, being the only set of patients that are not characterized by *EGFR* amplification. It displays a complex mix of genetic changes without dominance of any specific feature, all them in lower proportions than its counterparts cluster 2 and 3, and in a similar way as happens in the mesenchymal TCGA subtype [10,38]. On both sides, *EGFR* status-dependent subgroups of GBs, genetically and clinically different, can be separated. The identification of alterations in shared nodes of convergence downstream of RTKs hast been an interesting approach in cancer [4,8,58].

The present work sets out MLPA as an advantageous methodology, simple and useful for FFPE specimens. In addition, it may provide new insights into the molecular underpinnings of GB pathogenesis in a comprehensive manner. Clustering the genetic alterations of GB highlights the importance of *EGFR* in this very aggressive tumor type and could represent a strong step towards precision medicine: the aggregation of changes depending on the presence of the amplification of *EGFR*, the mutation variant III or both simultaneously, lead to different pathways to analyze. Our results underline the importance of *EGFR*vIII and *ADD3* SCNAs as markers of poor prognosis that need further consideration in GB. The presence of a group of GB-*IDH*wt without alterations in *EGFR* may explain part of the absence of effect of RTK inhibitors in this type of tumor. Furthermore, the clear separation of *EGFR*-amplified related GBs, showing sets of genes that are differentially altered, points to the need to rethink the possibilities of personalized therapy in future clinical settings. The differential groups that can be established could be used for a more accurate therapy.

## Figures and Tables

**Table 1:** Clinical data and baseline molecular characteristics of 137 glioblastoma patients.

Parameter		Specification	Outcome	Wild-Type IDH1/2 (n = 128)	Mutated IDH1 (n = 9)	*p*-Value
Age		Mean (range), in years	57.7 (24–81)	59 (24–81)	40.9 (32–52)	*** <0.001 ^mw^
		≤55	40.6%	36.1%	100%	*** <0.001 ^χ^2^^
		>55	59.4%	63.9%		
Sex		Male	54.0%	56.3%	22.2%	0.080 ^ft^
		Female	46.0%	43.7%	77.8%	
Tumor location		Parietal	35.0%	33.9%	50.0%	0.282 ^kw^
		Frontal	20.3%	19.1%	37.5%	
		Temporal	36.6%	38.3%	12.5%	
		Occipital	4.9%	5.2%		
		Intraventricular	0.8%	0.9%		
		Corpus Callosum	2.4%	2.6%		
Size (cm^3^)		Mean (range)	5.2 cm (2–11)	5.1 cm (2–11)	6.0 cm (5–7)	0.210 ^mw^
Initial symptom		Neurological deficit	30.0%	32.1%	0%	0.146 ^kw^
		Epileptic seizure	21.7%	21.4%	25.0%	
		Intracranial hypertension	48.3%	46.5%	75.0%	
KPS		≤85	76.9%	77.0%	75.0%	1.000 ^ft^
		>85	23.1%	23.0%	25.0%	
Overall survival		Median (95 CI)	210 days	180 days	3300 days	*** <0.001 ^lr^
*TP53*		Mutation	20.2%	17.3%	50.0%	* 0.028 ^χ^2^^
*EGFR* FISH		Alteration	61.4%	64.4%	22.2%	* 0.012 ^χ^2^^
		N-amp	38.6%	35.6%	77.8%	* 0.011 ^χ^2^^
		L-amp	13.4%	12.7%	22.2%	
		H-amp	48.0%	51.7%	0.0%	
*EGFR*vIII		No	65. 9%			
		Yes	34.1%			
SCNA	*EGFR*	Gain	65.4%	70.1%	0.0%	*** <0.001 ^χ^2^^
		Normal	34.6%	29.9%	100%	*** <0.001 ^kw^
	*CDKN2A*	Alteration	63.5%	65.6%	33.3%	0.052 ^χ^2^^
		Loss	53.3%			
		Gain	10.2%			
	*CDKN2B*	Alteration	54.0%	56.3%	22.2%	0.080 ^ft^
		Loss	48.9%			
		Gain	5.1%			
	*PTEN*	Alteration	65.9%	65.9%	66,7%	0.961 ^χ^2^^
		Loss	55.6%			
		Gain	10.4%			
	*MTAP*	Alteration	52.9%	53.5%	44.4%	0.734 ^ft^
	*TIMP3*	Alteration	65.0%	64.1%	77.8%	0.404 ^χ^2^^
	*ERBB2*	Alteration	26.5%			
	*MVP*	Alteration	59.5%	56.3%	100%	* 0.020 ^ft^
	*MEN1*	Alteration	60.6%	57.8%	100%	* 0.012 ^χ^2^^
	*ADD3*	Alteration	45.3%	46.9%	22.2%	0.183 ^ft^
	*PCCA*	Alteration	44.1%	46.6%	12.5%	0.075 ^ft^

Prevalence of genetic alterations and cross tabulation of IDH1 mutation status versus clinical characteristics and genetic alterations are depicted. For genetic alteration, only significant findings are shown (* means 0.05 > *p* > 0.01, ** means 0.01 > *p* > 0.001, *** means *p* < 0.001). Date are mean (range), number (%), or median (95%CI). Abbreviations: CNA, copy number alteration; ft, Fisher’s exact test; KFS, Karnofsky Performance Status; kw, Kruskal–Wallis test; lr, long-rank test; mw, Mann–Whitney–Wilcoxon test and χ^2^, Pearson’s chi-squared test.

**Table 2:** Data from TCGA analysis performed using cBioportal.

		*EGFR*			
		Shallow Deletion	Diploid	Gain	Amplification
*CDKN2A*	Deep deletion	2	20	131	178
	Shallow deletion	3	19	48	38
	Diploid	1	22	61	35
	Gain	0	2	14	3
	Amplification	0	1	0	0
	*** n = 577; *p* < 0.00001; Chi-square 461.258				
*JAG1*	Shallow deletion	1	3	13	1
	Diploid	3	52	153	126
	Gain	2	9	88	125
	Amplification	0	0	0	1
	*** n = 576; *p* < 0.00001; Chi-square 41.9092				
*MSH6*	Shallow deletion	0	8	15	14
	Diploid	4	55	220	225
	Gain	2	1	19	14
	n = 571; *p* > 0.05; Chi-square 7.2998				
*MTAP*	Deep deletion	2	18	114	169
	Shallow deletion	3	21	61	46
	Diploid	1	22	65	35
	Gain	0	2	14	3
	Amplification	0	1	0	0
	*** n = 570; *p* < 0.00001; Chi-square 46.413				

Annotated CNAs depending on *EGFR* amplification status. * was used for 0.050 > *p* > 0.010, ** for 0.010 *> p* > 0.001 and *** for *p* ≤ 0.001.

**Table 3:** Clustering analysis of GBM IDHwt.

Genes Studied	Cluster 1	Cluster 2	Cluster 3	*p*-Value
***CTNNB1***	11.1	8.0	28.6	0.120 (KW)
***CDH7***	0.0	12.0	4.8	0.166 (KW)
***IQGAP1***	18.5	12.0	23.8	0.581 (KW)
***GLDC***	7.4	12.0	23.8	0.254 (KW)
***TP53***	11.1	12.0	42.9	0.011 * (Chi)
***TGFBR2***	11.1	16.0	9.5	0.781 (KW)
***ING1***	18.5	24.0	33.3	0.495 (Chi)
***SIX3***	14.8	24.0	52.4	0.014 * (Chi)
***IL4***	14.8	24.0	66.7	<0.001 *** (Chi)
***PCCA***	44.4	24.0	85.7	<0.001 *** (Chi)
***DYSF***	18.5	28.0	42.9	0.180 (Chi)
***HNF1A***	37.0	28.0	57.1	0.124 (Chi)
***DOCK8***	7.4	32.0	9.5	0.036 * (KW)
***ERBB2***	14.8	36.0	19.0	0.169 (Chi)
***ATL1***	22.2	36.0	52.4	0.096 (Chi)
***ATR***	25.9	48.0	19.0	0.080 (Chi)
***TRAF4***	44.4	52.0	28.6	0.268 (Chi)
***JAG1***	3.7	56.0	9.5	<0.001 *** (Chi)
***SPG11***	14.8	56.0	9.5	<0.001 *** (Chi)
***EGFRvIII***	14.8	56.0	23.8	<0.001 *** (Chi)
***MSH6***	11.1	60.0	66.7	<0.001 *** (Chi)
***ADD3***	40.7	72.0	42.9	0.048 * (Chi)
***SMARCA4***	33.3	84.0	4.8	<0.001 *** (Chi)
***MVP***	51.9	84.0	14.3	<0.001 *** (Chi)
***MTAP***	22.2	84.0	76.2	<0.001 *** (Chi)
***PTEN***	44.4	88.0	38.1	<0.001 *** (Chi)
***TIMP3***	44.4	92.0	57.1	0.001 ** (Chi)
***EGFR***	40.7	92.0	85.7	<0.001 *** (Chi)
***MEN1***	44.4	96.0	23.8	<0.001 *** (Chi)
***CDKN2A***	7.4	100.0	100.0	<0.001 *** (Chi)

*p*-values were calculated by the (Chi), Pearson’s chi-squared test χ^2^ and (KW), Kruskal–Wallis test. To highlight statistical significance * was used for 0.050 > *p* > 0.010, ** for 0.010 *> p* > 0.001 and *** for *p* ≤ 0.001.

## Supplementary Materials

The following are available online at https://www.mdpi.com/2073-4409/9/11/2429/s1, Table S1: Amplification status of *EGFR* determined by interphase Fluorescence in situ hybridization and MLPA analysis.

## References

[1] Louis D.N., Perry A., Reifenberger G., von Deimling A., Figarella-Branger D., Cavenee W.K., Ohgaki H., Wiestler O.D., Kleihues P., Ellison D.W. (2016). The 2016 World Health Organization Classification of Tumors of the Central Nervous System: A summary. Acta Neuropathol..

[2] Ostrom Q.T., Gittleman H., Liao P., Vecchione-Koval T., Wolinsky Y., Kruchko C., Barnholtz-Sloan J.S. (2017). CBTRUS Statistical Report: Primary brain and other central nervous system tumors diagnosed in the United States in 2010–2014. Neuro Oncol..

[3] Lawrence M.S., Stojanov P., Mermel C.H., Robinson J.T., Garraway L.A., Golub T.R., Meyerson M., Gabriel S.B., Lander E.S., Getz G. (2014). Discovery and saturation analysis of cancer genes across 21 tumour types. Nature.

[4] Furnari F.B., Cloughesy T.F., Cavenee W.K., Mischel P.S. (2015). Heterogeneity of epidermal growth factor receptor signalling networks in glioblastoma. Nat. Rev. Cancer.

[5] Karsy M., Gelbman M., Shah P., Balumbu O., Moy F., Arslan E. (2012). Established and emerging variants of glioblastoma multiforme: Review of morphological and molecular features. Folia Neuropathol..

[6] Lu J., Cowperthwaite M.C., Burnett M.G., Shpak M. (2016). Molecular predictors of long-term survival in glioblastoma multiforme patients. PLoS ONE.

[7] Umehara T., Arita H., Yoshioka E., Shofuda T., Kanematsu D., Kinoshita M., Kodama Y., Mano M., Kagawa N., Fujimoto Y. (2019). Distribution differences in prognostic copy number alteration profiles in IDH-wild-type glioblastoma cause survival discrepancies across cohorts. Acta Neuropathol. Commun..

[8] Gerlinger M., Rowan A.J., Horswell S., Math M., Larkin J., Endesfelder D., Gronroos E., Martinez P., Matthews N., Stewart A. (2012). Intratumor heterogeneity and branched evolution revealed by multiregion sequencing. N. Engl. J. Med..

[9] Hochberg F.H., Atai N.A., Gonda D., Hughes M.S., Mawejje B., Balaj L., Carter R.S. (2014). Glioma diagnostics and biomarkers: An ongoing challenge in the field of medicine and science. Expert Rev. Mol. Diagn..

[10] Verhaak R.G.W., Hoadley K.A., Purdom E., Wang V., Qi Y., Wilkerson M.D., Miller C.R., Ding L., Golub T., Mesirov J.P. (2010). An integrated genomic analysis identifies clinically relevant subtypes of glioblastoma characterized by abnormalities in PDGFRA, IDH1, EGFR and NF1. Cancer Cell.

[11] Ohgaki H., Kleihues P. (2009). Genetic alterations and signaling pathways in the evolution of gliomas. Cancer Sci..

[12] Cancer Genome Atlas Research Network (2008). The Cancer Genome Atlas Research Network Comprehensive genomic characterization defines human glioblastoma genes and core pathways. Nature.

[13] Huse J.T. (2014). Elucidating the oncogenic role of ATRX deficiency in glioma. Neuro Oncol..

[14] Ohgaki H., Kleihues P. (2007). Genetic pathways to primary and secondary glioblastoma. Am. J. Pathol..

[15] Sahm F., Reuss D.E., Giannini C. (2018). WHO 2016 classification: Changes and advancements in the diagnosis of miscellaneous primary CNS tumours. Neuropathol. Appl. Neurobiol..

[16] Lassman A.B., Roberts-Rapp L., Sokolova I., Song M., Pestova E., Kular R., Mullen C., Zha Z., Lu X., Gomez E. (2019). Comparison of biomarker assays for EGFR: Implications for precision medicine in patients with glioblastoma. Clin. Cancer Res..

[17] Anderson M.G., Falls H.D., Mitten M.J., Oleksijew A., Vaidya K.S., Boghaert E.R., Gao W., Palma J.P., Cao D., Chia P.-L. (2020). Targeting Multiple EGFR-expressing Tumors with a Highly Potent Tumor-selective Antibody-Drug Conjugate. Mol. Cancer Ther..

[18] Kim G., Ko Y.T. (2020). Small molecule tyrosine kinase inhibitors in glioblastoma. Arch. Pharm. Res..

[19] Lopez-Gines C., Gil-Benso R., Ferrer-Luna R., Benito R., Serna E., Gonzalez-Darder J., Quilis V., Monleon D., Celda B., Cerdá-Nicolas M. (2010). New pattern of EGFR amplification in glioblastoma and the relationship of gene copy number with gene expression profile. Modern Pathol..

[20] Stichel D., Ebrahimi A., Reuss D., Schrimpf D., Ono T., Shirahata M., Reifenberger G., Weller M., Hänggi D., Wick W. (2018). Distribution of EGFR amplification, combined chromosome 7 gain and chromosome 10 loss, and TERT promoter mutation in brain tumors and their potential for the reclassification of IDHwt astrocytoma to glioblastoma. Acta Neuropathol..

[21] Navarro L., Gil-Benso R., Megías J., Muñoz-Hidalgo L., San-Miguel T., Callaghan R.C., González-Darder J.M., López-Ginés C., Cerdá-Nicolás M.J. (2015). Alteration of major vault protein in human glioblastoma and its relation with EGFR and PTEN status. Neuroscience.

[22] An Z., Aksoy O., Zheng T., Fan Q.-W., Weiss W.A. (2018). Epidermal growth factor receptor (EGFR) and EGFRvIII in glioblastoma (GBM): Signaling pathways and targeted therapies. Oncogene.

[23] Parker J.J., Canoll P., Niswander L., Kleinschmidt-DeMasters B.K., Foshay K., Waziri A. (2018). Intratumoral heterogeneity of endogenous tumor cell invasive behavior in human glioblastoma. Sci. Rep..

[24] Muñoz-Hidalgo L., San-Miguel T., Megías J., Monleón D., Navarro L., Roldán P., Cerdá-Nicolás M., López-Ginés C. (2020). Somatic copy number alterations are associated with EGFR amplification and shortened survival in patients with primary glioblastoma. Neoplasia.

[25] Pedersen M.W., Tkach V., Pedersen N., Berezin V., Poulsen H.S. (2004). Expression of a naturally occurring constitutively active variant of the epidermal growth factor receptor in mouse fibroblasts increases motility. Int. J. Cancer.

[26] Feng H., Hu B., Vuori K., Sarkaria J.N., Furnari F.B., Cavenee W.K., Cheng S.-Y. (2014). EGFRvIII stimulates glioma growth and invasion through PKA-dependent serine phosphorylation of Dock180. Oncogene.

[27] Keller S., Schmidt M.H.H. (2017). EGFR and EGFRvIII promote angiogenesis and cell invasion in glioblastoma: Combination therapies for an effective treatment. Int. J. Mol. Sci..

[28] Boisselier B., Dugay F., Belaud-Rotureau M.-A., Coutolleau A., Garcion E., Menei P., Guardiola P., Rousseau A. (2018). Whole genome duplication is an early event leading to aneuploidy in IDH-wild type glioblastoma. Oncotarget.

[29] Mao X., Hamoudi R.A. (2000). Molecular and cytogenetic analysis of glioblastoma multiforme. Cancer Genet. Cytogenet..

[30] Jeuken J., Cornelissen S., Boots-Sprenger S., Gijsen S., Wesseling P. (2006). Multiplex Ligation-Dependent Probe Amplification. J. Mol. Diagn..

[31] Jeuken J., Sijben A., Alenda C., Rijntjes J., Dekkers M., Boots-Sprenger S., McLendon R., Wesseling P. (2009). Robust Detection of EGFR Copy Number Changes and EGFR Variant III: Technical Aspects and Relevance for Glioma Diagnostics. Brain Pathol..

[32] González J.R., Carrasco J.L., Armengol L., Villatoro S., Jover L., Yasui Y., Estivill X. (2008). Probe-specific mixed-model approach to detect copy number differences using multiplex ligation-dependent probe amplification (MLPA). BMC Bioinform..

[33] Gessi M., Hammes J., Lauriola L., Dörner E., Kirfel J., Kristiansen G., zur Muehlen A., Denkhaus D., Waha A., Pietsch T. (2013). GNA11 and N-RAS mutations: Alternatives for MAPK pathway activating GNAQ mutations in primary melanocytic tumours of the central nervous system. Neuropathol. Appl. Neurobiol..

[34] Gessi M., Gielen G.H., Denkhaus D., Antonelli M., Giangaspero F., zur Mühlen A., Japp A.S., Pietsch T. (2015). Molecular heterogeneity characterizes glioblastoma with lipoblast/adipocyte-like cytology. Virchows Arch..

[35] Gan H.K., Kaye A.H., Luwor R.B. (2009). The EGFRvIII variant in glioblastoma multiforme. J. Clin. Neurosci..

[36] Layfield L., Willmore C., Tripp S., Jones C., Jensen R. (2006). Epidermal growth factor receptor gene amplification and protein expression in glioblastoma multiforme. Appl. Immunohistochem. Mol. Morphol..

[37] Cerami E., Gao J., Dogrusoz U., Gross B.E., Sumer S.O., Aksoy B.A., Jacobsen A., Byrne C.J., Heuer M.L., Larsson E. (2012). The cBio Cancer Genomics Portal: An open platform for exploring multidimensional cancer genomics data. Cancer Discov..

[38] Brennan C.W., Verhaak R.G.W., McKenna A., Campos B., Noushmehr H., Salama S.R., Zheng S., Chakravarty D., Sanborn J.Z., Berman S.H. (2013). The Somatic Genomic Landscape of Glioblastoma. Cell.

[39] Szklarczyk D., Gable A.L., Lyon D., Junge A., Wyder S., Huerta-Cepas J., Simonovic M., Doncheva N.T., Morris J.H., Bork P. (2019). STRING v11: Protein–protein association networks with increased coverage, supporting functional discovery in genome-wide experimental datasets. Nucleic Acids Res..

[40] Ciriello G., Sinha R., Hoadley K.A., Jacobsen A.S., Reva B., Perou C.M., Sander C., Schultz N. (2013). The molecular diversity of Luminal A breast tumors. Breast Cancer Res. Treat..

[41] Crespo I., Vital A.L., Nieto A.B., Rebelo O., Tão H., Lopes M.C., Oliveira C.R., French P.J., Orfao A., Tabernero M.D. (2011). Detailed characterization of alterations of chromosomes 7, 9, and 10 in glioblastomas as assessed by single-nucleotide polymorphism arrays. J. Mol. Diagn..

[42] Sahin A., Sanchez C., Bullain S., Waterman P., Weissleder R., Carter B.S. (2018). Development of third generation anti-EGFRvIII chimeric T cells and EGFRvIII-expressing artificial antigen presenting cells for adoptive cell therapy for glioma. PLoS ONE.

[43] Kiang K.M.-Y., Leung G.K.-K. (2018). A review on Adducin from functional to pathological mechanisms: Future direction in cancer. BioMed Res. Int..

[44] van den Boom J., Wolter M., Kuick R., Misek D.E., Youkilis A.S., Wechsler D.S., Sommer C., Reifenberger G., Hanash S.M. (2003). Characterization of gene expression profiles associated with glioma progression using oligonucleotide-based microarray analysis and real-time reverse transcription-polymerase chain reaction. Am. J. Pathol..

[45] Huang H., Colella S., Kurrer M., Yonekawa Y., Kleihues P., Ohgaki H. (2000). Gene expression profiling of low-grade diffuse astrocytomas by cDNA arrays. Cancer Res..

[46] Mariani L., Beaudry C., McDonough W.S., Hoelzinger D.B., Demuth T., Ross K.R., Berens T., Coons S.W., Watts G., Trent J.M. (2001). Glioma cell motility is associated with reduced transcription of proapoptotic and proliferation genes: A cDNA microarray analysis. J. Neurooncol..

[47] Kiang K.M.-Y., Zhang P., Li N., Zhu Z., Jin L., Leung G.K.-K. (2020). Loss of cytoskeleton protein ADD3 promotes tumor growth and angiogenesis in glioblastoma multiforme. Cancer Lett..

[48] Lechuga S., Amin P.H., Wolen A.R., Ivanov A.I. (2019). Adducins inhibit lung cancer cell migration through mechanisms involving regulation of cell-matrix adhesion and cadherin-11 expression. Biochim. Biophys. Acta Mol. Cell Res..

[49] Sun Q., Pei C., Li Q., Dong T., Dong Y., Xing W., Zhou P., Gong Y., Zhen Z., Gao Y. (2018). Up-regulation of MSH6 is associated with temozolomide resistance in human glioblastoma. Biochem. Biophys. Res. Commun..

[50] Skiriute D., Vaitkiene P., Saferis V., Asmoniene V., Skauminas K., Deltuva V.P., Tamasauskas A. (2012). MGMT, GATA6, CD81, DR4, and CASP8 gene promoter methylation in glioblastoma. BMC Cancer.

[51] Hansen L.J., Sun R., Yang R., Singh S.X., Chen L.H., Pirozzi C.J., Moure C.J., Hemphill C., Carpenter A.B., Healy P. (2019). MTAP Loss Promotes Stemness in Glioblastoma and Confers Unique Susceptibility to Purine Starvation. Cancer Res..

[52] Lubin M., Lubin A. (2009). Selective killing of tumors deficient in methylthioadenosine phosphorylase: A novel strategy. PLoS ONE.

[53] Marjon K., Cameron M.J., Quang P., Clasquin M.F., Mandley E., Kunii K., McVay M., Choe S., Kernytsky A., Gross S. (2016). MTAP Deletions in Cancer Create Vulnerability to Targeting of the MAT2A/PRMT5/RIOK1 Axis. Cell Rep..

[54] Tang B., Lee H.-O., An S.S., Cai K.Q., Kruger W.D. (2018). Specific Targeting of MTAP-Deleted Tumors with a Combination of 2′-Fluoroadenine and 5′-Methylthioadenosine. Cancer Res..

[55] Kesarwani P., Prabhu A., Kant S., Chinnaiyan P. (2019). Metabolic Remodeling Contributes Towards an Immune Suppressive Phenotype in Glioblastoma. Cancer Immunol. Immunother..

[56] Ciaglia E., Abate M., Laezza C., Pisanti S., Vitale M., Seneca V., Torelli G., Franceschelli S., Catapano G., Gazzerro P. (2017). Antiglioma effects of N6-isopentenyladenosine, an endogenous isoprenoid end product, through the downregulation of epidermal growth factor receptor. Int. J. Cancer.

[57] Ciaglia E., Grimaldi M., Abate M., Scrima M., Rodriquez M., Laezza C., Ranieri R., Pisanti S., Ciuffreda P., Manera C. (2017). The isoprenoid derivative N6-benzyladenosine CM223 exerts antitumor effects in glioma patient-derived primary cells through the mevalonate pathway. Br. J. Pharmacol..

[58] Qiu X.-X., Chen L., Wang C.-H., Lin Z.-X., Chen B.-J., You N., Chen Y., Wang X.-F. (2016). The Vascular Notch Ligands Delta-Like Ligand 4 (DLL4) and Jagged1 (JAG1) Have Opposing Correlations with Microvascularization but a Uniform Prognostic Effect in Primary Glioblastoma: A Preliminary Study. World Neurosurg..

