# Supplementary material for: Identification of New Genetic Clusters in Glioblastoma Multiforme: EGFR Status and ADD3 Losses Influence Prognosis
Source: Cells. 2020 Nov 6;9(11):2429. doi: 10.3390/cells9112429 (PMC7694764; doi:10.3390/cells9112429)
Supplement: Supplementary file 1 [file cells-09-02429-s001.pdf]

Supplementary table 1. Amplification status of EGFR determined by interphase Fluorescence *in situ* hybridization and MLPA analysis.

	EGFR STATUS			EGFR STATUS			EGFR STATUS			EGFR STATUS	
**Case**	**FISH**	**MLPA**	**Case**	**FISH**	**MLPA**	**Case**	**FISH**	**MLPA**	**Case**	**FISH**	**MLPA**
1	N-AMP	N-AMP	33	N-AMP	N-AMP	65	N-AMP	GAIN	97	H-AMP	GAIN
2	N-AMP	N-AMP	34	N-AMP	N-AMP	66	H-AMP	GAIN	98	H-AMP	GAIN
3	N-AMP	N-AMP	35	na	N-AMP	67	N-AMP	GAIN	99	H-AMP	GAIN
4	N-AMP	N-AMP	36	N-AMP	N-AMP	68	N-AMP	GAIN	100	H-AMP	GAIN
5	N-AMP	N-AMP	37	L-AMP	N-AMP	69	H-AMP	GAIN	101	H-AMP	GAIN
6	N-AMP	N-AMP	38	L-AMP	N-AMP	70	N-AMP	GAIN	102	H-AMP	GAIN
7	N-AMP	N-AMP	39	H-AMP	GAIN	71	L-AMP	GAIN	103	H-AMP	GAIN
8	L-AMP	N-AMP	40	na	GAIN	72	N-AMP	GAIN	104	H-AMP	GAIN
9	N-AMP	N-AMP	41	N-AMP	GAIN	73	H-AMP	GAIN	105	H-AMP	GAIN
10	N-AMP	N-AMP	42	N-AMP	GAIN	74	L-AMP	GAIN	106	H-AMP	GAIN
11	N-AMP	N-AMP	43	N-AMP	GAIN	75	L-AMP	GAIN	107	H-AMP	GAIN
12	N-AMP	N-AMP	44	L-AMP	GAIN	76	H-AMP	GAIN	108	H-AMP	GAIN
13	N-AMP	N-AMP	45	na	GAIN	77	H-AMP	GAIN	109	H-AMP	GAIN
14	Na	N-AMP	46	N-AMP	GAIN	78	L-AMP	GAIN	110	H-AMP	GAIN
15	N-AMP	N-AMP	47	N-AMP	GAIN	79	H-AMP	GAIN	111	H-AMP	GAIN
16	N-AMP	N-AMP	48	N-AMP	GAIN	80	H-AMP	GAIN	112	H-AMP	GAIN
17	L-AMP	N-AMP	49	H-AMP	GAIN	81	H-AMP	GAIN	113	H-AMP	GAIN
18	N-AMP	N-AMP	50	na	GAIN	82	H-AMP	GAIN	114	H-AMP	GAIN
19	Na	N-AMP	51	L-AMP	GAIN	83	H-AMP	GAIN	115	H-AMP	GAIN
20	N-AMP	N-AMP	52	na	GAIN	84	H-AMP	GAIN	116	H-AMP	GAIN
21	Na	N-AMP	53	N-AMP	GAIN	85	H-AMP	GAIN	117	H-AMP	GAIN
22	N-AMP	N-AMP	54	N-AMP	GAIN	86	H-AMP	GAIN	118	H-AMP	GAIN
23	Na	N-AMP	55	L-AMP	GAIN	87	H-AMP	GAIN	119	H-AMP	GAIN
24	N-AMP	N-AMP	56	L-AMP	GAIN	88	H-AMP	GAIN	120	H-AMP	GAIN
25	N-AMP	N-AMP	57	L-AMP	GAIN	89	H-AMP	GAIN	121	H-AMP	GAIN
26	N-AMP	N-AMP	58	N-AMP	GAIN	90	H-AMP	GAIN	122	H-AMP	GAIN
27	N-AMP	N-AMP	59	H-AMP	GAIN	91	H-AMP	GAIN	123	H-AMP	GAIN
28	N-AMP	N-AMP	60	L-AMP	GAIN	92	H-AMP	GAIN	124	H-AMP	GAIN
29	Na	N-AMP	61	N-AMP	GAIN	93	H-AMP	GAIN	125	H-AMP	GAIN
30	H-AMP	N-AMP	62	H-AMP	GAIN	94	H-AMP	GAIN	126	H-AMP	GAIN
31	N-AMP	N-AMP	63	L-AMP	GAIN	95	H-AMP	GAIN	127	H-AMP	GAIN
32	N-AMP	N-AMP	64	H-AMP	GAIN	96	H-AMP	GAIN	128	H-AMP	GAIN

Abbreviations: H-AMP, high level of EGFR copies; L-AMP, low level of EGFR copies; N-AMP, no EGFR amplification in iFISH column/ copy number detection ratio between 0.7 and 1.3 in MLPA column; GAIN, copy number detection ratio above x: ≥1.3; na, no available data.
